# Unveiling the role of dorsal root ganglia in spasticity reduction: Insights from contralateral seventh cervical nerve cross transfer surgery

**DOI:** 10.1002/brb3.3613

**Published:** 2024-07-05

**Authors:** Xuanyu Zhao, Xingyi Ma, Huali Zhao, Tie Li, Yanqun Qiu, Yundong Shen, Juntao Feng, Wendong Xu

**Affiliations:** ^1^ Department of Hand and Upper Extremity Surgery, Jing'an District Central Hospital, Branch of Huashan Hospital, the National Clinical Research Center for Aging and Medicine Fudan University Shanghai China; ^2^ Department of Radiology, Jing'an District Central Hospital, Branch of Huashan Hospital, the National Clinical Research Center for Aging and Medicine Fudan University Shanghai China; ^3^ Institute of Brain Science, State Key Laboratory of Medical Neurobiology and Collaborative Innovation Center for Brain Science Fudan University Shanghai China; ^4^ Research Unit of Synergistic Reconstruction of Upper and Lower Limbs after Brain Injury Chinese Academy of Medical Sciences Shanghai China; ^5^ Co‐innovation Center of Neuroregeneration Nantong University Nantong China

**Keywords:** C7 nerve root neurotomy, contralateral seventh cervical nerve cross transfer, dorsal root ganglion, spasticity

## Abstract

**Background:**

Central nervous system (CNS) disorders, such as stroke, often lead to spasticity, which result in limb deformities and significant reduction in quality of life. Spasticity arises from disruptions in the normal functioning of cortical and descending inhibitory pathways in the brainstem, leading to abnormal muscle contractions. Contralateral seventh cervical nerve cross transfer (CC7) surgery has been proven to effectively reduce spasticity, but the specific mechanism for its effectiveness is unclear.

**Methods:**

This study aimed to investigate the changes in the dorsal root ganglia (DRG) following CC7 surgery. A comprehensive anatomical analysis was conducted through cadaveric study and magnetic resonance imaging (MRI) study, to accurately measure the regional anatomy of the C7 DRG. DRG perfusion changes were quantitatively assessed by comparing pre‐ and postoperative dynamic contrast‐enhanced (DCE) MRI.

**Results:**

In CC7 surgery, the C7 nerve root on the affected side is cut close to the DRG (3.6 ± 1.0 mm), while the C7 nerve root on the healthy side is cut further away from the DRG (65.0 ± 10.0 mm). MRI studies revealed that after C7 proximal neurotomy on the affected side, there was an increase in DRG volume, vascular permeability, and perfusion; after C7 distal neurotomy on the healthy side, there was a decrease in DRG volume, with no significant changes in vascular permeability and perfusion.

**Conclusion:**

This study provides preliminary insights into the mechanisms of spasticity reduction following CC7 surgery, indicating that changes in the DRG, such as increased vascular permeability and perfusion, could disrupt abnormal spinal *γ*‐circuits. The resulting high‐perfusion state of DRG, possibly due to heightened neuronal activity and metabolic demands, necessitating further research to verify this hypothesis.

## Graphical abstract

This study investigated the dorsal root ganglia (DRG) changes following contralateral seventh cervical nerve cross transfer (CC7) surgery, a procedure used to treat spasticity. Our results showed that the proximity of the section site of the C7 nerve root to the DRG induced an inflammatory response, leading to changes in the DRG morphology, which may contribute to the reduction of spasticity following CC7 surgery. This study provides insights into the underlying mechanisms of spasticity reduction and highlights the importance of the DRG in the effectiveness of CC7 surgery.

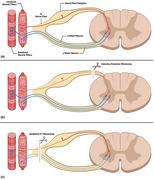

## 1 INTRODUCTION

Central nervous system (CNS) disorders affect over 100 million individuals worldwide, with an annual incidence surpassing 10 million cases (Collaborators, 2021). Spasticity is a prevalent consequence of CNS disorders, leading to limb deformities and a significant decline in quality of life. CNS disorders disrupt the normal functioning of cortical and descending inhibitory pathways in the brainstem (Gras & Leclercq, 2017; S. Li et al., 2021), resulting in reduced γ‐motor neuron inhibition, heightened muscle spindle sensitivity (Marrone et al., 2022; Peacock & Arens, 1982), and abnormal discharges. Consequently, α‐motor neurons, influenced by afferent fibers from the muscle spindles, manifest spastic muscle contractions characterized by spasms, tremors, or stiffness (Schalow & Zäch, 1996).

The dorsal root ganglia (DRG) is integral in conveying sensory signals related to various sensory modalities and is involved in modulating spasms through the spinal cord γ‐circuit (Enslin et al., 2019), where a group of γ‐motor neurons, upon receiving signals from the brain in the normal circuit, initiate a series of reflex arcs that facilitate precise and coordinated muscle contractions (Bishop, 1977). The DRG has been used as a therapeutic target for spasticity in patients with cerebral palsy and spinal cord injury (Chang & Cho, 2017; De Louw et al., 2002; Vles et al., 2010), shedding light on the research of the underlying mechanism of reducing spasticity (Guo et al., 2012).

In 2011, Xu was the first to perform contralateral C7 transfer surgery for the treatment of upper limb spastic paralysis after cerebral palsy (Xu et al., 2011). Subsequently, in 2018, contralateral seventh cervical nerve cross transfer (CC7) was utilized for patients with spastic arm paralysis. This surgical procedure involves the relocation of the C7 nerve from the healthy side to re‐establish a connection between the paralyzed limb and the non‐paralyzed hemisphere (Zheng et al., 2018). Clinical observations have shown that CC7 surgery results in an immediate reduction in spasticity across the entire upper limb, including the elbow, forearm, wrist, and fingers, extending beyond the area directly innervated by the C7 nerve (Feng et al., 2022). The specific mechanism responsible for the reduction in spasticity following CC7 surgery remains uncertain.

CC7 surgery involves neurotomy of the C7 nerves on each side at different distances from the DRGs. The C7 neurotomy near the intervertebral foramen, termed the proximal neurotomy, is hypothesized to induce changes in the DRG. It is postulated that these DRG changes, along with the disruption of the abnormal spinal cord γ‐circuit, contribute to the reduction of spasticity following CC7 surgery in the early stage. To investigate this hypothesis, our study utilized both cadaveric and magnetic resonance imaging (MRI) techniques for mutual validation to obtain local anatomical data on C7 DRG and to measure the distance between the neurotomy site of the bilateral C7 nerve roots and the DRG in CC7 surgery. Additionally, preoperative and 3‐month postoperative dynamic contrast‐enhanced (DCE) MRI images were compared to assess the morphological and perfusion changes in the bilateral DRGs.

## 2 MATERIALS AND METHODS

### 2.1 Cadaveric study

Seventeen sides of 10 unembalmed fresh adult cadavers were randomly selected for this study, including four females and six males. The cadaveric study was performed at the Department of Anatomy, Histology, and Embryology, Shanghai Medical College, Fudan University. Cadavers with cervical congenital dysplasia or a history of surgical trauma to the neck were excluded from the study. This study was conducted in accordance with the Declaration of Helsinki.

**FIGURE 1:**
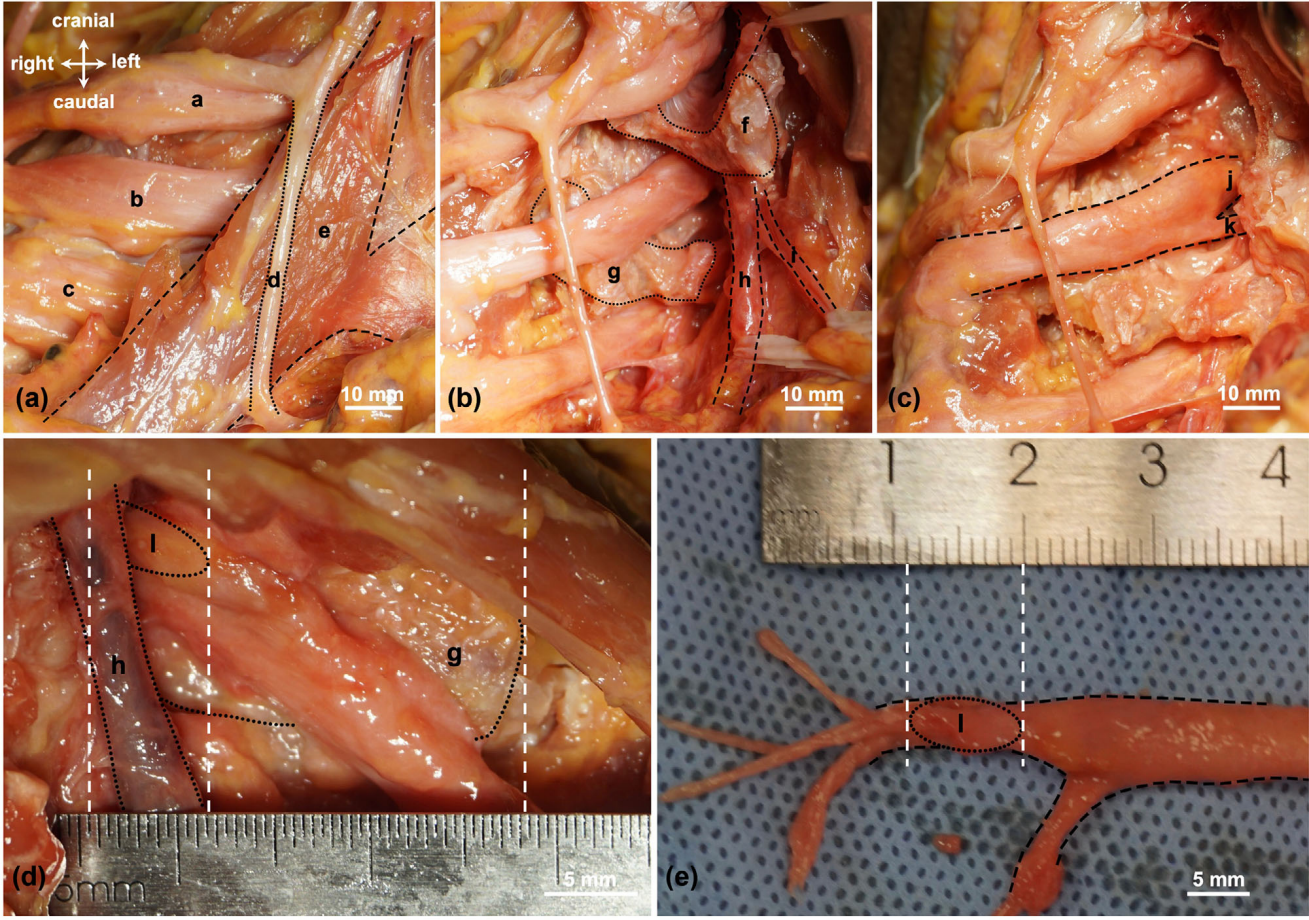
Anatomical operation of fresh adult cadavers. (a) The exposed anterior scalene muscle, phrenic nerve, and brachial plexus. (b) The anterior and middle scalene muscles were cut off to expose the vertebral artery and vein. (c) The C6 transverse process was bitten off to expose the anterior and posterior roots of the C7 spinal nerve. (d) Measurement of the anatomical relationship between C7 DRG, the vertebral artery, and the transverse process. The distal end of C7 DRG is located 6 mm from the vertebral artery. (e) Measurement of diameter of the thickest part and the length of C7 DRG. The C7 DRG measures 9 mm in length. a: superior trunk of brachial plexus, b: middle trunk of brachial plexus (C7 nerve root), c: inferior trunk of brachial plexus, d: phrenic nerve, e: anterior scalene muscle, f: anterior tubercle of the C6 transverse process, g: transverse process of the seventh cervical vertebra, h: vertebral artery, i: vertebral vein, j: C7 dorsal root, k: C7 anterior root, and l: C7 DRG.

The cadaver was placed in the supine position, and a longitudinal incision was made at the posterior edge of the sternocleidomastoid muscle to expose the anterior scalene muscle, phrenic nerve, and brachial plexus (Figure 1a). The insertions of the anterior scalene muscle and part of the longus neck muscle were cut off at the anterior tubercle of the sixth cervical vertebra. The vertebral artery was exposed to record the relationship between the vertebral artery and the brachial plexus. The insertion of the middle scalene muscle was detached at the posterior tubercles of the sixth and seventh cervical vertebrae. The vertebral artery traversed the C6 transverse process foramen and did not pass through the C7 foramen (Figure 1b). A rongeur was used to remove the C6 transverse process, completely exposing the vertebral artery and the distal end of the DRG (Figure 1c). An osteotome and rongeur were used to remove the lateral border of the sixth cervical vertebra to reveal the proximal end of the DRG (Figure 1d). Vernier calipers measured the anatomic parameters of the DRG, including the diameter and length of the C7 DRG and the distance from its distal end to the C7 transverse process (TP) (Figure 1e).

### 2.2 MRI study

#### 2.2.1 Patients and imaging data

The MRI study was divided into three parts; a total of 20 normal subjects and 25 patients were included in this study. The first part was to obtain neuroanatomical parameters of the C7 DRG by brachial plexus MRI images of 20 normal subjects. The second part involved MRI examinations of 15 patients before and 3 months after CC7 surgery, to assess the extent of neurotomy and the volume changes in the C7 DRGs. For the third part, DCE MRI was performed on 10 patients before and after surgery to compare pre‐ and postoperative MRI signal intensity and blood‐tissue permeability K^trans^.

We chose to perform MRI scan 3 months postoperatively because the bleeding caused by surgical trauma had ceased, and the changes in the DRG due to inflammation had stabilized by this time. The new neural circuit established by the CC7 surgery requires 12 months to fully form; therefore, 3 months post‐surgery is considered the early postoperative phase.

**FIGURE 2:**
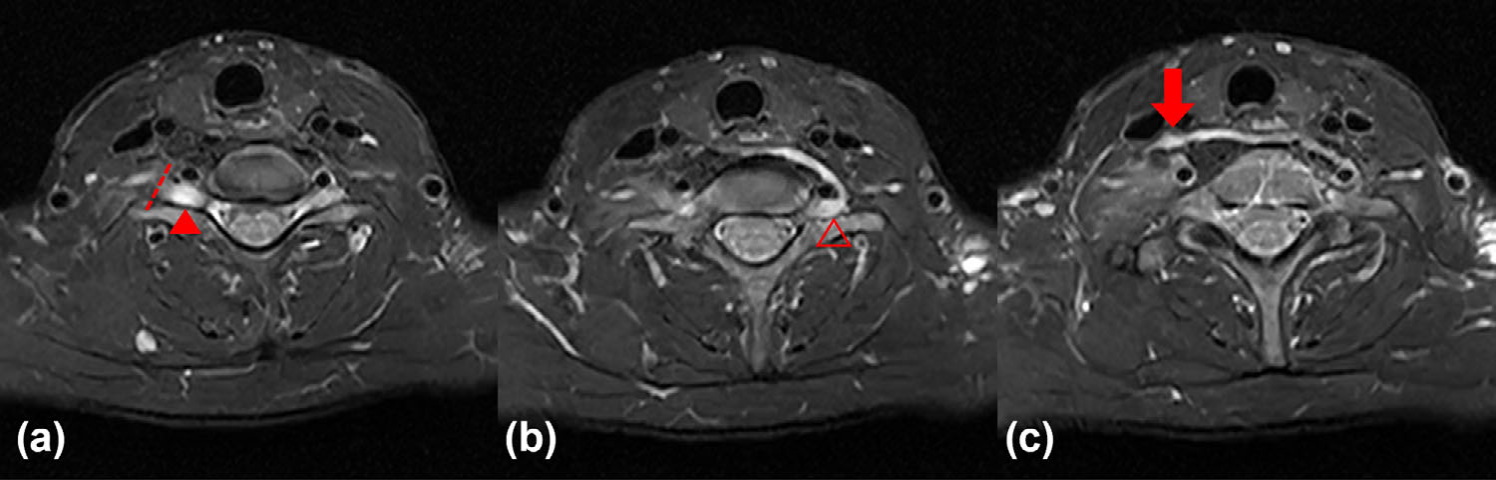
Axial T2‐weighted magnetic resonance imaging (MRI) images of the patient after CC7 surgery in different cross‐section. (a) Dashed line indicates the section position of the C7 nerve root on the paralyzed side near the intervertebral foramen, solid triangle represents the paralyzed side's dorsal root ganglia (DRG). (b) Hollow triangle represents the nonparalyzed side's DRG. It can be observed that the DRG on the affected side has a significantly increased volume and MRI signal intensity compared to the DRG on the healthy side. (c) Arrow indicates the site of nerve anastomosis after CC7 surgery.

All postoperative patients underwent standardized Xu's CC7 procedure (Zheng et al., 2018). During CC7 surgery, surgeons section the C7 nerve root on the paralyzed side proximal to intervertebral foramen (Figure 2a), while sectioning the C7 nerve root on the nonparalyzed side distally before it converges with other brachial plexus nerves (Figure 2b,c). This allows for a sufficient nerve length, and the two ends are then connected through an anterior cervical approach.

Inclusion criteria: patients with spasticity in the upper extremity caused by CNS injury such as stroke, traumatic brain injury, cerebral palsy, and encephalitis; patients who have had symptoms for >1 year; patients intending to undergo CC7 surgery to improve the function of the upper limb; patients who are older than 18 years of age; patients of any gender; and patients in stable mental health.

This study was approved by the Institutional Review Board of Jing'an District Central Hospital (2022‐22) and registered in the Chinese clinical trial registry (ChiCTR2200062345). All experiments on human subjects were conducted in accordance with the Declaration of Helsinki, and all procedures were carried out with the adequate understanding and written consent of the subjects.

#### 2.2.2 Imaging preprocessing

**FIGURE 3:**
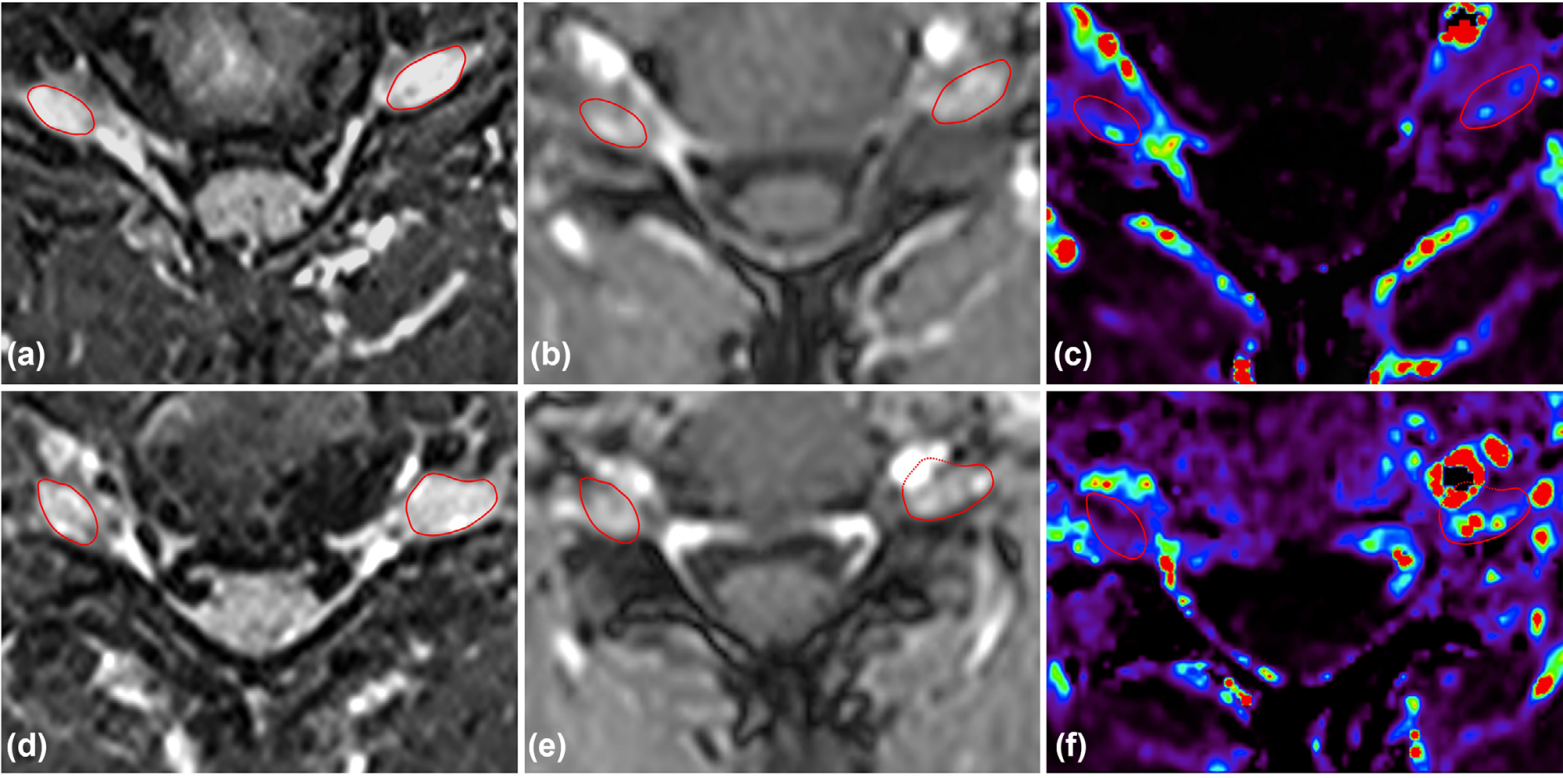
Perfusion magnetic resonance imaging (MRI) images of C7 dorsal root ganglia (DRG). (a–c) T2‐weighed images, gadolinium‐enhanced T1‐weighted images, and pseudo‐color dynamic contrast‐enhanced (DCE) MRI images of C7 DRG in preoperative patients. (d–f) T2‐weighed images, gadolinium‐enhanced T1‐weighted images, and pseudo‐color DCE MRI images of C7 DRG in postoperative patients, right side was the paralyzed side after section. The C7 DRG was circled by red lines. Both the volume and the vascular perfusion of the C7 DRG on the affected side are higher than those on the opposite side.

For this study, we used China United Imaging 1.5T uMR660 MRI. During scanning, the patients were in the supine position, with a head neck coil and a chest abdominal coil. The scanning range was from the skull base plane to the lower edge plane of the fourth thoracic spine. Axial and coronal sequences of the C7 DRG were acquired. The scanned sequences included, t1_fse_tra (FOV: 220 mm, thickness/interval: 1.4/0.1 mm, resolution: 288 × 85, TR: 777 ms, TE: 10 ms) and t2_fse_wfi_tra (FOV: 220 mm, thickness/interval: 1.8/0.1 mm, resolution: 240 × 80, TR: 2595 ms, TE: 85.8 ms, Figure 3a,d).

A T1‐weighted, DCE volumetric‐interpolated breath‐hold examination sequence (repetition time/echo time 4.67/2.18 ms, flip angle 12°, 24 slices, resolution 1.48 × 1.19 × 6.00 mm^3^) covering the neck from the upper edge of the sixth cervical vertebra to the lower edge of the first thoracic vertebra. Before contrast agent administration, T1‐weighted volumetric interpolated breath‐hold examination sequences with 2°, 6°, 9°, and 12° flip angles were acquired (Figure 3b,e). The contrast agent was administered intravenously via automated injection at the beginning of the third frame of the sequence, using a standard concentration of 0.1 mmol kg^−1^ and a flow rate of 3.0 mL s^−1^. A total of 24 frames were recorded at a rate of 9.1 s frame^−1^. An eight‐channel receive/transmit spine coil and an eight‐channel receive body flex coil (united imaging) were used.

### 2.3 Measurements

The diameter was measured at the widest position of the DRG. The length was measured at the expansion segment of the C7 spinal nerve dorsal root. The distance between the distal end of the DRG and the distal end of the seventh cervical transverse process (posterior tubercle) was measured. From MRI images, the distance between the distal end of the DRG and the neurotomy position of the C7 nerve root was measured. The volume of the DRG was estimated with the following approximation (Godel et al., 2017): volume = (horizontal × sagittal × coronal diameter)/2.

To further investigate the structural aspects of the DRG, T2‐weighted MRI with a higher signal was performed to reflect vascular changes resulting from neuroinflammation after neurotomy (Chhabra et al., 2018). Image masks were obtained by manual segmentation around the contours of the bilateral C7 DRGs, which were reliably visible in the anatomical, T2‐weighted nonenhanced images (Godel et al., 2016). DCE MRI scans were utilized to obtain the K^trans^ perfusion parameter of the DRG, enabling quantitative analysis of perfusion changes (Godel et al., 2017). T2‐weighted MRI signal intensity and blood‐tissue permeability K^trans^ maps of the DRG and spinal nerves were generated and quantitatively assessed using 10 preoperative and 10 postoperative DCE MRI images (Figure 3c,f).

Three observers (a hand surgeon, a hand surgery fellow, and a senior resident) took measurements independently, employing a blind and randomized approach. The three observers’ measurements were averaged and considered to be the final value.

### 2.4 Statistical analysis

Contrasting DRG volume differences between anatomical measurements and MRI can optimize sensitivity (West et al., 2012). The measurements are presented as the means ± standard deviations and 95% confidence intervals. The group means were compared using *t*‐tests. Differences were considered significant at *p* < .05. All the data were analyzed by SPSS 20.

## 3 RESULTS

### 3.1 Anatomical and MRI measurements of DRG

**FIGURE 4:**
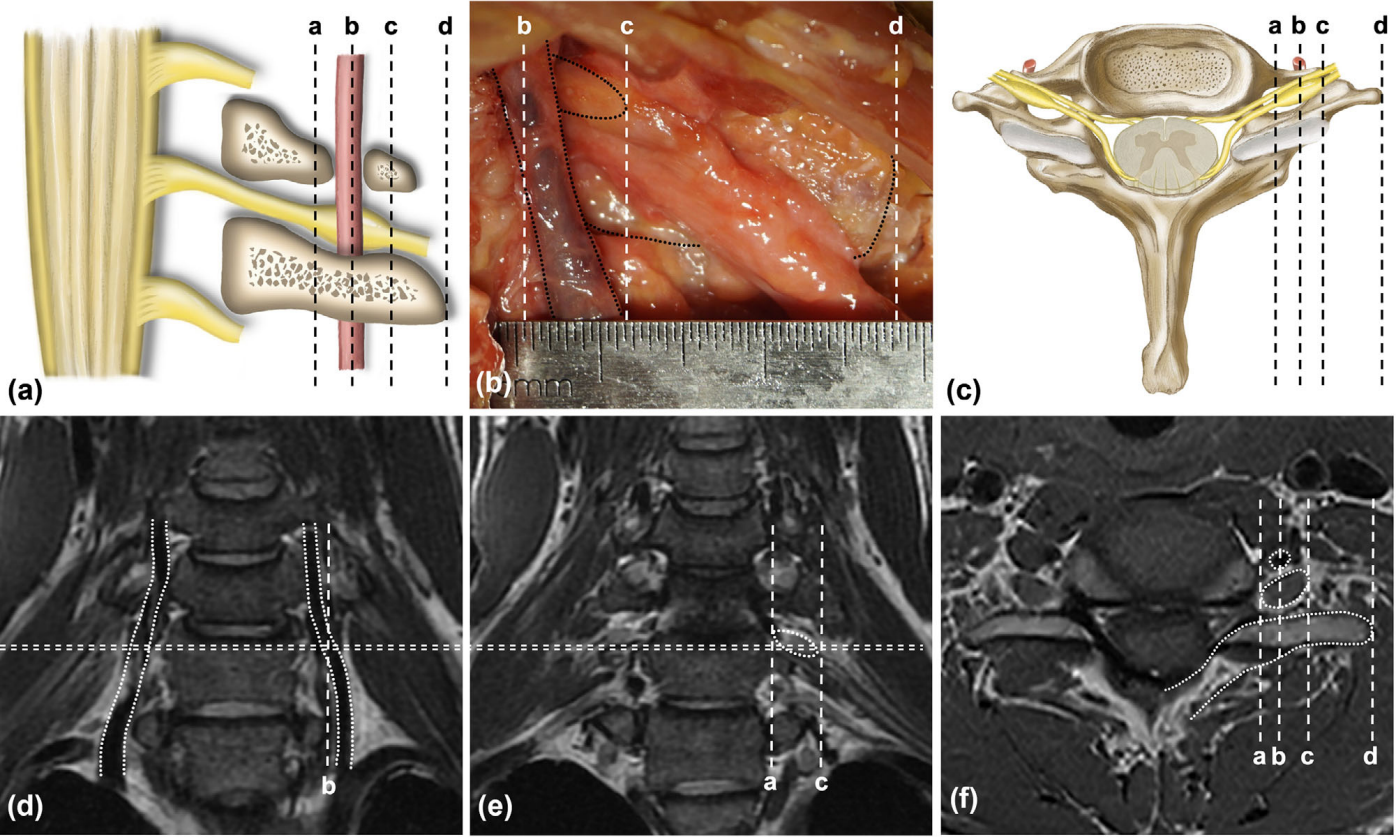
Measurement of dorsal root ganglia (DRG)‐related anatomical structure. (a) Schematic diagram of C7 DRG and surrounding anatomical structures in coronal view. (b) Coronal view of fresh cadaver. (c) Schematic diagram of C7 DRG and surrounding anatomical structures in axial view. (d) Coronal magnetic resonance imaging (MRI) image of the brachial plexus, the vertebral artery was marked by dotted line. (e) Coronal MRI image of the brachial plexus, C7 DRG was marked by dotted line. (f) Axial MRI image of the brachial plexus, the vertebral artery, C7 DRG, and transverse process were marked by dotted line. Dashed line a: proximal end of C7 DRG. Dashed line b: vertebral artery. Dashed line c: distal end of C7 DRG. Dashed line d: distal end of C7 transverse process.

**FIGURE 5:**
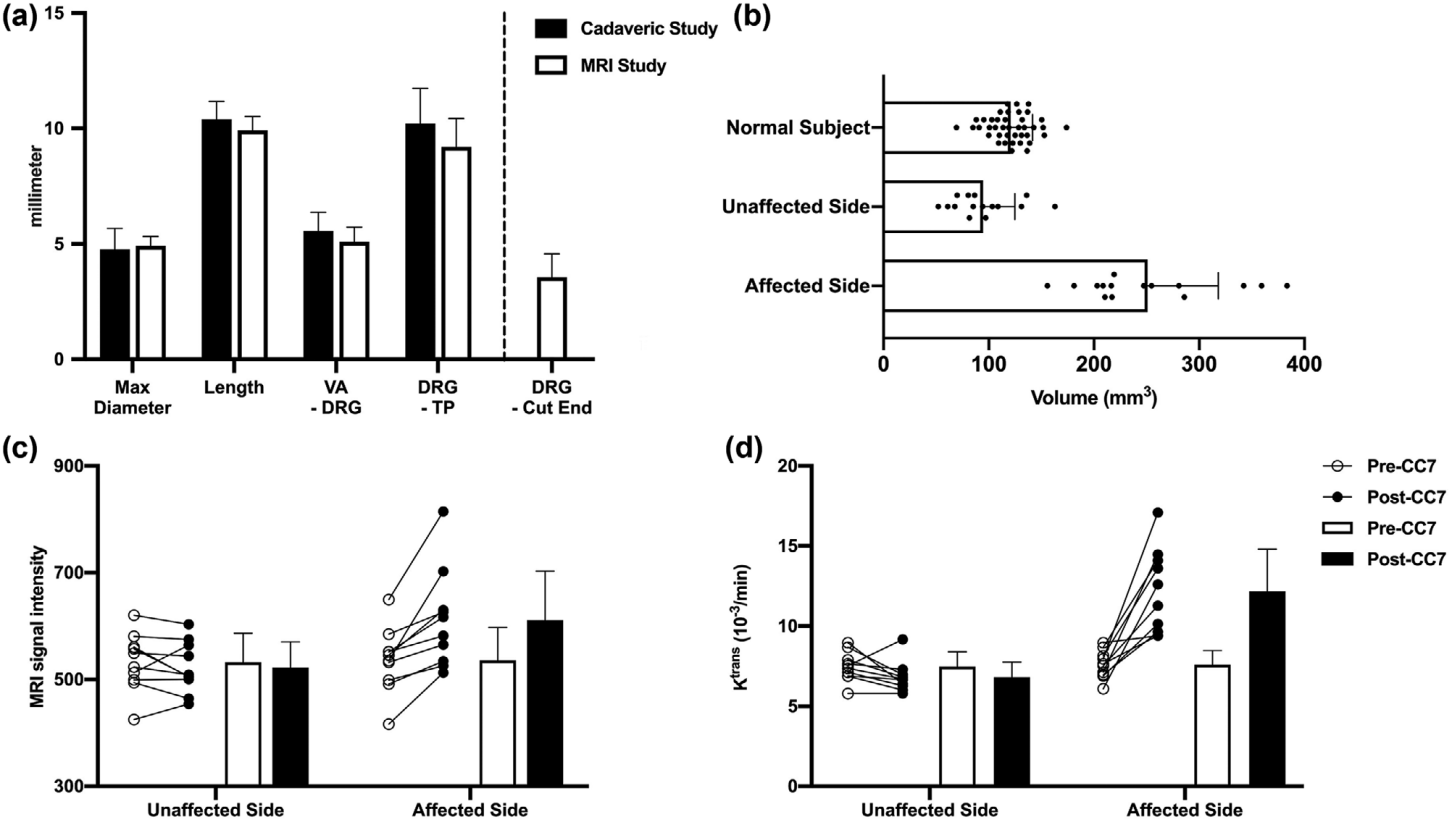
Statistical results. (a) Statistical map of the comparison between cadaveric and magnetic resonance imaging (MRI) study, and the distance from section to the distal end of C7 dorsal root ganglia (DRG) in MRI images. There was no significant difference in the measurements between the cadaveric and MRI studies (*p* > .05). The neurotomy site of the C7 nerve root on the paralyzed side was located 3.6 ± 1.0 mm away from the DRG. (b) Changes of DRG volume after CC7 surgery. The preoperative DRG volume was 120.8 ± 20.7 mm^3^. The volume of DRG on the paralyzed side (neurotomy adjacent to the DRG) significantly increased to 251.0 ± 67.0 mm^3^ (*p* < .001), while DRG volume on the non‐paralyzed side (neurotomy distant from the DRG) significantly decreased to 94.5 ± 30.2 mm^3^ (*p* < .001). (c) Changes of T2‐weighed MRI signal intensity in C7 DRG after CC7 surgery. On the unaffected side, it changed from 532.4 ± 54.1 to 522.7 ± 48.1 (*p* > .05); on the affected side, it changed from 536.0 ± 61.0 to 611.1 ± 91.9 (*p* < .001). (d) Changes of perfusion parameter (K^trans^) in C7 DRG after CC7 surgery. On the unaffected side, it changed from 7.5 ± 0.9 10^−3^ min^−1^ to 6.8 ± 0.9 10^−3^ min^−1^ (*p* > .05); on the affected side, it changed from 7.6 ± 0.9 10^−3^ min^−1^ to 12.2 ± 2.6 10^−3^ min^−1^ (*p* < .001). TP, transverse process; VA, vertebral artery.

In the cadaveric study, the morphological size of the DRG was measured by a calliper, revealing a maximum diameter of 4.8 ± 0.9 mm and a length of 10.4 ± 0.8 mm (Figure 4a,c), consistent with previous literature findings (Leng et al., 2018). The distance from the distal neurotomy site of the DRG to the distal site of the transverse process (TP) was 10.2 ± 1.5 mm (Figures 4b and 5a). In the MRI study of normal subjects, the maximum diameter of the DRG was 4.9 ± 0.4 mm, the length of the DRG was 9.9 ± 0.6 mm (Figure 4e), and the distance from the distal site of the DRG to the distal site of the TP was 9.2 ± 1.2 mm (Figures 4d,f and 5A). There was no significant difference in the neuroanatomical measurements between the cadaveric and MRI studies (*p* > .05). The MRI studies of postoperative patients showed that the proximal section site of the C7 nerve root was located 3.6 ± 1.0 mm beyond the DRG, while the distance of the distal C7 nerve root section site was 65.0 ± 10.0 mm.

### 3.2 Volumetric changes in the DRG

The pre‐ and postoperative MRI images of 15 patients were categorized based on the condition of each side into three groups: normal (preoperative), non‐paralyzed (postoperative and distal‐cut), and paralyzed (postoperative and proximal‐cut) sides. The preoperative DRG volume was 120.8 ± 20.7 mm^3^; the postoperative DRG volume on the paralyzed side increased to 251.0 ± 67.0 mm^3^ (*p* < .001), and the postoperative DRG volume on the nonparalyzed side decreased to 94.5 ± 30.2 mm^3^ (*p* < .001; Figure 5b).

### 3.3 T2‐weighted MRI signal intensity and permeability of DRG

Upon comparing the pre‐ and postoperative DCE MRI images of 10 patients, the average T2‐weighted MRI signal intensity of the C7 DRG increased from 536.0 ± 61.0 to 611.1 ± 91.9 (*p* < .001) following proximal C7 neurotomy on the paralyzed side; conversely, it decreased from 532.4 ± 54.1 to 522.7 ± 48.1 (*p* > .05; Figure 5c) after distal neurotomy on the nonparalyzed side. After the proximal neurotomy, the C7 DRG exhibited significantly increased permeability as K^trans^ changed from 7.6 ± 0.9 10^−3^ min^−1^ to 12.2 ± 2.6 10^−3^ min^−1^ (*p* < .001); however, the K^trans^ of the C7 DRG following distal neurotomy changed from 7.5 ± 0.9 10^−3^ min^−1^ to 6.8 ± 0.9 10^−3^ min^−1^ (*p* > .05, Figure 5d).

## 4 DISCUSSION

Clinical trials and neurobiological studies have provided compelling evidence for the effectiveness of CC7 nerve transfer in treating spastic hemiparesis resulting from CNS injury (Alawieh et al., 2021; Feng et al., 2022; Ratican et al., 2021; Xu, 2020; Yu et al., 2019). C7 nerve holds a unique role in its connection with both the superior and inferior trunks of the brachial plexus, collectively supplying innervation to the entire upper extremity (Gesslbauer et al., 2017). The re‐establishment of peripheral nerve pathways after CC7 surgery may contribute to long‐term spasticity reduction, possibly associated with changes in the white matter fiber tracts of the corticospinal tract and corpus callosum (Lee et al., 2021). However, the reasons behind the changes in spasticity during the early postoperative period remain unclear. As reported in a multicenter real‐world study involving 425 patients (Feng et al., 2022), 3 months after CC7 surgery, there was a significant reduction in the Modified Ashworth Scale scores for the entire upper limb (including the elbow, forearm, wrist, and fingers), and improvements in upper limb motor function had already started to appear. Clearly, 3 months is insufficient to complete the re‐establishment of all neural circuits in the upper limb; therefore, the significant early postoperative improvements are likely attributable solely to the neurotomy of the C7 nerve root close to the distal end of the DRG.

**FIGURE 6:**
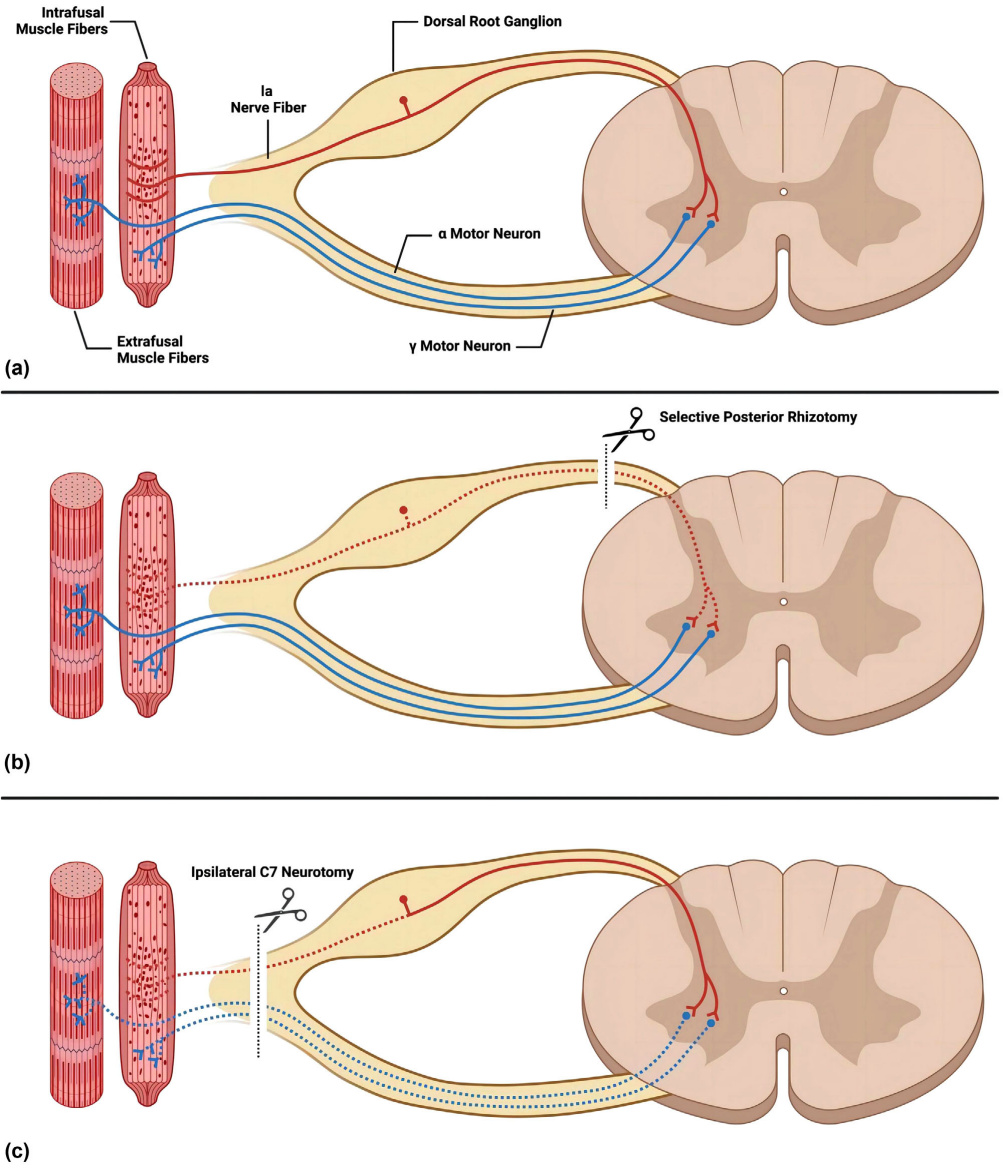
Spinal circuit with sensory nerve fiber and motor neuron. (a) Normal spinal circuit from muscle spindle to muscle fiber through connection between peripheral motor system and central nerve system. (b) Section of the posterior root was performed in selective posterior rhizotomy to prevent sensory input. (c) Sensory input and motor output were prevented by ipsilateral (the paralyzed side) C7 neurotomy, which is adjacent to the dorsal root ganglia (DRG).

Selective posterior rhizotomy (SPR) surgery, a classical approach to treating spasticity (Bertelli et al., 2000; Enslin et al., 2019; Maarrawi et al., 2006), interrupts the connection between the dorsal root ramus and the CNS (Enslin et al., 2019) (Figure 6a). The mechanism involves manipulating the spinal nerves within the vertebral bodies and interrupting aberrant excitatory signal transmission by targeting the γ‐circuit (Seruya, 2018) (Figure 6b). However, cervical SPR to relieve the muscle spasticity of the upper limb may have relatively high surgical risk, because the cervical spinal cord connects the brain and other segments, and its function is dense and important.

During CC7 surgery, the procedure involves sectioning the cervical nerve roots that emanate from the intervertebral foramen, which contain both motor and sensory fibers, without touching the vertebral body (Figure 6c). Additionally, CC7 surgery may effectively blocks abnormal motor and sensory signaling within the γ‐circuit on the affected side (Bai et al., 2022), resulting in an immediate reduction of spasticity without significant functional impairment. SPR surgery focuses on interrupting the connection between the dorsal sensory root branch of the spinal cord and the CNS to address spasticity. It involves the sectioning of the ramus, a filiform structure of the posterior root nerve, located within the intervertebral foramen. Therefore, CC7 surgery demonstrates superiority in alleviating spasticity while preserving functional integrity.

Most existing studies on the DRG focus predominantly on its involvement in pathological neuralgia, emphasizing solely its sensory function while disregarding its broader interactions within the spinal nerve circuit. Consequently, this has constrained the research, overlooking the upstream and downstream connections within the circuit. Additionally, there is a notable lack of targeted investigations into the anatomical and functional aspects of DRG concerning surgical interventions. DRG is an enlarged structure located near the intervertebral foramen along the posterior roots of the spinal cord. There is one DRG within each intervertebral foramen that contains a significant number of primary sensory neurons. The DRG serves as a crucial junction linking the peripheral and CNSs, with peripheral neurosurgery exerting a profound influence on inducing CNS remodeling. As an active participant in the neural circuitry, the DRG plays a vital role in transmitting information about the external and internal environments of the human body to the CNS (Delrée et al., 1993; Neumann & Woolf, 1999; Neumann et al., 2002). By elucidating the anatomical characteristics of the DRG and the alterations in the DRG following neurotomy, a deeper understanding of the neuroscientific mechanisms underlying CC7 surgery can be attained, particularly regarding how modifications in peripheral nerve pathways can induce central remodeling.

This is the first study to investigate the local anatomical structure of C7 DRG through cadaveric research and compare it with MRI results, providing important anatomical references for CC7 surgery and laying the foundation for future mechanistic research. In this anatomical study, we simulated CC7 surgery on fresh cadavers and compared the cadaveric results with in vivo brachial plexus MRI data. There were no significant differences in the size and specific location of the DRG between the cadaveric anatomy and the MRI imaging results, which validated each other to ensure the reliability of our research. The distances from the proximal neurotomy site of the C7 nerve root to the DRG were measured to be 3.6 ± 1.0 mm (very close to DRG), while those from the distal neurotomy were 65.0 ± 10.0 mm (far from DRG).

DRG neurons, characterized as pseudo‐unipolar, have non‐regenerative central branch axons, while their peripheral branches demonstrate robust regenerative capacity. By severing the C7 nerve root at the DRG and allowing the migration of the proximal C7 nerve root, a clinical scenario akin to postganglionic brachial plexus injury is replicated. This intentional reduction of presynaptic sensory input creates an artificial peripheral nerve injury, enabling targeted modulation of the intrinsic state of DRG neurons and facilitating the potential for central neuronal regeneration.

This study makes the first investigation into the DRG changes following CC7 surgery, analyzing MRI data of patients both preoperatively and 3 months postoperatively. After cutting the C7 nerve root on the paralyzed side close to the DRG (3.6 ± 1.0 mm), there was an increase in DRG volume, vascular permeability, and perfusion; after cutting the C7 nerve root on the healthy side far from the DRG (65.0 ± 10.0 mm), there was a decrease in DRG volume and no significant change in perfusion. This aligns with the notion that the neurotomy close to the DRG leads to an inflammatory response in nerve root fibers, potentially altering the DRG neurons and their networks (C. L. Li et al., 2016), thus affecting the spastic state.

DRG is an intensely vascularized organ, characterized by a dense capillary network (Jimenez‐Andrade et al., 2008). The employment of DCE‐MRI facilitated the distinction between various microstructural compartments within the DRG (Godel et al., 2016). Three months post‐surgery, patients have recovered from the trauma and inflammation caused by the surgical procedure and tend toward a more stable state. The data collected at this time point represent changes in DRG microvascular perfusion following inflammation. Our study findings highlight postoperative increases in vascular permeability and perfusion of the C7 DRG on the affected side. We speculate that this high‐perfusion state of the C7 DRG caused by neurotomy is related to the alleviation of spasms. While the increase in DRG volume and perfusion may suggest enhanced neuronal activity, current data are insufficient to directly confirm a correlation with increased metabolic demand. Future research should involve more detailed fundamental studies to clarify this hypothesis.

This study revealed different changes of DRG following proximal and distal C7 neurotomy in CC7 surgery for upper limb spastic paralysis, as evidenced by anatomical and MRI imaging study. However, the results were limited by using the 1.5T MRI, which affects imaging quality. Additionally, this study was limited to patients 3 months postoperative; it did not encompass longer postoperative durations. The study provides valuable insights into the underlying mechanisms behind the reduction of spasticity following CC7 surgery, particularly emphasizing the changes in DRGs and the complete sectioning of the abnormal spinal cord γ‐circuit. Further exploration into the correlation between upper extremity spasticity management and DRG changes could lead to a better understanding of CC7 surgery for spastic paralysis.

In future studies, it is essential to employ 3.0T or higher field strength MRI and refine MRI parameters to enhance imaging quality. This improved methodology will facilitate a more detailed analysis of the DRG changes in brachial plexus beyond C7, thereby enabling a comprehensive understanding of the overall changes. Additionally, incorporating more time points into longitudinal studies is crucial to effectively track the timeline of inflammatory changes following surgery. Given the unclear mechanism of inflammation in C7 nerve root neurotomy, investigating the effects of anti‐inflammatory treatments on DRG changes could provide valuable confirmatory evidence. Based on the findings of this article, further histological studies are also needed to gain a deeper understanding of the functions of the DRG after increases in volume and perfusion.

## AUTHOR CONTRIBUTIONS

**Xuanyu Zhao**: Conceptualization; data curation; formal analysis; investigation; methodology; software; validation; visualization; writing—original draft; writing—review and editing. **Xingyi Ma**: Conceptualization; investigation; validation; writing—original draft; writing—review and editing. **Huali Zhao**: Conceptualization; data curation; formal analysis; methodology; software; validation; visualization. **Tie Li**: Investigation; methodology; resources. **Yanqun Qiu**: Investigation; methodology; resources. **Yundong Shen**: Investigation; methodology; project administration; resources; supervision. **Juntao Feng**: Investigation; project administration; supervision; validation. **Wendong Xu**: Conceptualization; funding acquisition; investigation; project administration; resources; supervision; validation; writing—review and editing.

## CONFLICT OF INTEREST STATEMENT

The authors declare no conflicts of interest.

### PEER REVIEW

The peer review history for this article is available at https://publons.com/publon/10.1002/brb3.3613.

## Data Availability

The data that support the findings of this study are available from the corresponding author upon reasonable request.
